# A pneumatic power harvesting ankle-foot orthosis to prevent foot-drop

**DOI:** 10.1186/1743-0003-6-19

**Published:** 2009-06-16

**Authors:** Robin Chin, Elizabeth T Hsiao-Wecksler, Eric Loth, Géza Kogler, Scott D Manwaring, Serena N Tyson, K Alex Shorter, Joel N Gilmer

**Affiliations:** 1 Department of Mechanical Science and Engineering, University of Illinois at Urbana-Champaign, 1206 West Green Street, Urbana, Illinois 61801, USA; 2 Department of Aerospace Engineering, University of Illinois at Urbana-Champaign, 104 South Wright Street, Urbana, Illinois 61801, USA; 3 Clinical Biomechanics Laboratory, School of Applied Physiology, Georgia Institute of Technology, 281 Ferst Drive, Atlanta, Georgia 30332, USA

## Abstract

**Background:**

A self-contained, self-controlled, pneumatic power harvesting ankle-foot orthosis (PhAFO) to manage foot-drop was developed and tested. Foot-drop is due to a disruption of the motor control pathway and may occur in numerous pathologies such as stroke, spinal cord injury, multiple sclerosis, and cerebral palsy. The objectives for the prototype PhAFO are to provide toe clearance during swing, permit free ankle motion during stance, and harvest the needed power with an underfoot bellow pump pressurized during the stance phase of walking.

**Methods:**

The PhAFO was constructed from a two-part (tibia and foot) carbon composite structure with an articulating ankle joint. Ankle motion control was accomplished through a cam-follower locking mechanism actuated via a pneumatic circuit connected to the bellow pump and embedded in the foam sole. Biomechanical performance of the prototype orthosis was assessed during multiple trials of treadmill walking of an able-bodied control subject (n = 1). Motion capture and pressure measurements were used to investigate the effect of the PhAFO on lower limb joint behavior and the capacity of the bellow pump to repeatedly generate the required pneumatic pressure for toe clearance.

**Results:**

Toe clearance during swing was successfully achieved during all trials; average clearance 44 ± 5 mm. Free ankle motion was observed during stance and plantarflexion was blocked during swing. In addition, the bellow component repeatedly generated an average of 169 kPa per step of pressure during ten minutes of walking.

**Conclusion:**

This study demonstrated that fluid power could be harvested with a pneumatic circuit built into an AFO, and used to operate an actuated cam-lock mechanism that controls ankle-foot motion at specific periods of the gait cycle.

## Background

Foot-drop is a condition where the foot does not effectively clear the ground due to weak or absent ankle dorsiflexors which results in a steppage-type gait pattern. Steppage gait is a compensatory walking pattern for foot-drop that is characterized by increased knee and hip flexion during the swing phase to insure that the toe clears the ground during walking. The cause of foot-drop can be neurological and/or muscular in origin due to a multitude of pathologies [1]. A common treatment intervention is the use of an ankle-foot orthosis (AFO) that supports the ankle and foot to preclude foot-drop. This study presents a novel self-contained power harvesting ankle-foot orthosis that controls the unwanted plantarflexion movements associated with foot-drop and permits free ankle motion during stance.

The incidence of foot-drop is difficult to determine since it is a symptom rather than a disease; therefore numerous pathologies may present with foot-drop as a confounding factor. Essentially foot-drop is due to a disruption of the motor control pathway that occurs in the brain, peroneal nerve, spinal cord and/or muscle. Some of the common conditions that may present with a foot-drop are: trauma, incomplete spinal cord injuries, stroke, multiple sclerosis, muscular dystrophies and cerebral palsy.

An effective orthosis should only provide the biomechanical controls necessary to improve the functional deficit without a perturbing effect to other normal movements and functions. Charcot-Marie-Tooth (CMT) disease is an inherited neurological disorder that typically presents with weak dorsiflexors and associated foot-drop [2]. As the plantarflexors are frequently not affected, the ideal foot-drop AFO for CMT should permit free ankle plantarflexion (with mild resistance) and dorsiflexion during stance phase and block plantarflexion during swing, i.e., prevent foot-drop.

Many current AFO designs used for foot-drop treatment are effective at controlling the undesirable plantarflexion during swing, but do not permit free ankle motion during the stance phase of gait [3,4]. In conventional metal and leather systems, metal hinge joints with various types of stops are used to block motion while springs serve to resist or assist movement. The posterior leaf spring ankle foot orthosis is a plastic shell that encompasses the posterior and plantar aspect of the leg and foot respectively, with the distal half of the shank section acting as a flexible strut (the leaf spring). These AFO's do not have articulated ankle joints and instead are dependent upon the material properties and geometry which determine motion control characteristics. Because the thermoplastics (i.e. polypropylene) used in these AFO strut designs have a resistive property when they are deformed, their biomechanical motion control is restricted to resistive and assistive functions and cannot permit free movement. Hybrid AFO systems take advantage of the light weight plastic shell designs but are equipped with an articulated ankle joint to have a better anatomical/mechanical joint alignment and provide more diverse motion control options (i.e. stops, assists etc.). However, the drawback of these AFO designs is that the motion control elements cannot be timed and operated to different periods of the gait cycle. Therefore many of the motion control functions are present at times that they are not needed or even inhibit desirable motion. A relatively small and efficient spring and oil damper ankle joint was recently developed by Yamamoto and colleagues [5,6] to resist plantarflexion at initial contact and limit plantarflexion during swing. These studies demonstrated that an AFO outfitted with an oil damper ankle joint effectively improved gait compared to conventional mechanical ankle joint systems. However, the authors acknowledged that further research was needed to develop AFO's that could provide their own power while ensuring compactness and portability.

Using external power supplies, orthoses equipped with computer-driven powered actuators have demonstrated that specific motion-control tasks can be carried out to influence functional parameters of gait with relatively precise accuracy, e.g. [7-14]. However the applicability of these orthoses is limited to laboratory- and clinical-based studies since off-board power supplies and computers are required for their operation, i.e., these are tethered systems. To respond to this need, an AFO has been developed herein which includes a self-harvesting power system to control a mechanical ankle joint with a miniature actuator. The control features of the system provide toe clearance during swing and allow free ankle motion during the stance phase of gait.

A novel aspect of the proposed Power-harvesting Ankle-Foot Orthosis (PhAFO) is the capacity to re-generate power through the compression of a bellow located in the sole of the foot component. The pneumatic (air) power system is designed to be charged during mid to late stance of each gait cycle. The stored pneumatic power is designed to drive an actuated cam-lock mechanism that prevents plantarflexion of the foot during swing. At initial heel contact, a touch-valve (air pressure release-valve) located at the posterior plantar surface of the AFO is activated. This valve dispels air through an exhaust port which disengages the lock to allow free movement of the ankle and foot during stance phase. Thus, the need for an external power source and controller for the AFO can be eliminated.

To test the biomechanical effects of the PhAFO during walking, a pilot study was conducted on an unimpaired healthy control subject. The objectives of this pilot study were to determine if the PhAFO could: (1) repeatedly harvest fluid power during mid to late stance during walking, (2) use this power to actuate a cam-lock mechanism at specific periods of the gait cycle, (3) provide adequate toe clearance during swing, and (4) permit free ankle foot motion during stance.

## Methods

### Ankle foot orthosis

**Figure 1:**
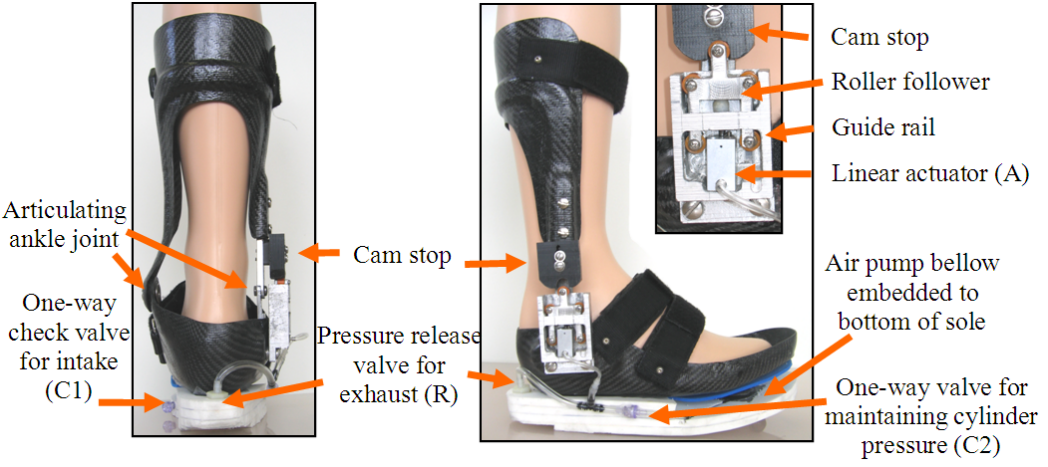
**Posterior and lateral views of the power-harvesting AFO (PhAFO)**. See Figure 2 for reference to symbols (A, R, C1, C2).

The current prototype was designed as a stand-alone device, not intended for insertion into a shoe (Fig. 1). The PhAFO was comprised of two major components, a posterior shell tibial section and a foot piece. Both parts were custom fabricated out of a pre-impregnated carbon composite laminate material over a positive model of a leg. The foot shell (US men's size 11) incorporated a shoe-last profile that placed the heel 1.0 cm higher with respect to the metatarsal heads. The toe section of the foot plate was oriented, at a five degree angle (pitch) relative to the ground, to emulate late stance rollover since the foot section was rigid and did not allow natural dorsiflexion of the phalanges. Conventional free motion ankle hinge joints connected the foot piece and tibial section. Velcro straps were used to secure the PhAFO to the leg and foot. The actuator and cam-lock control mechanism were attached to the lateral aspect of the AFO. A stationary cam was attached to the lateral upright of the tibial section and an actuated roller-follower mechanism was affixed to the foot piece. A foam sole was cemented to the plantar surface of the foot shell. The device and attachments have a total mass of 1 kg. (Conventional foot-drop AFOs weigh between 300–600 g; however since these designs require insertion into a shoe, the weights of the PhAFO and conventional AFO plus shoe are comparable.)

**Figure 2:**
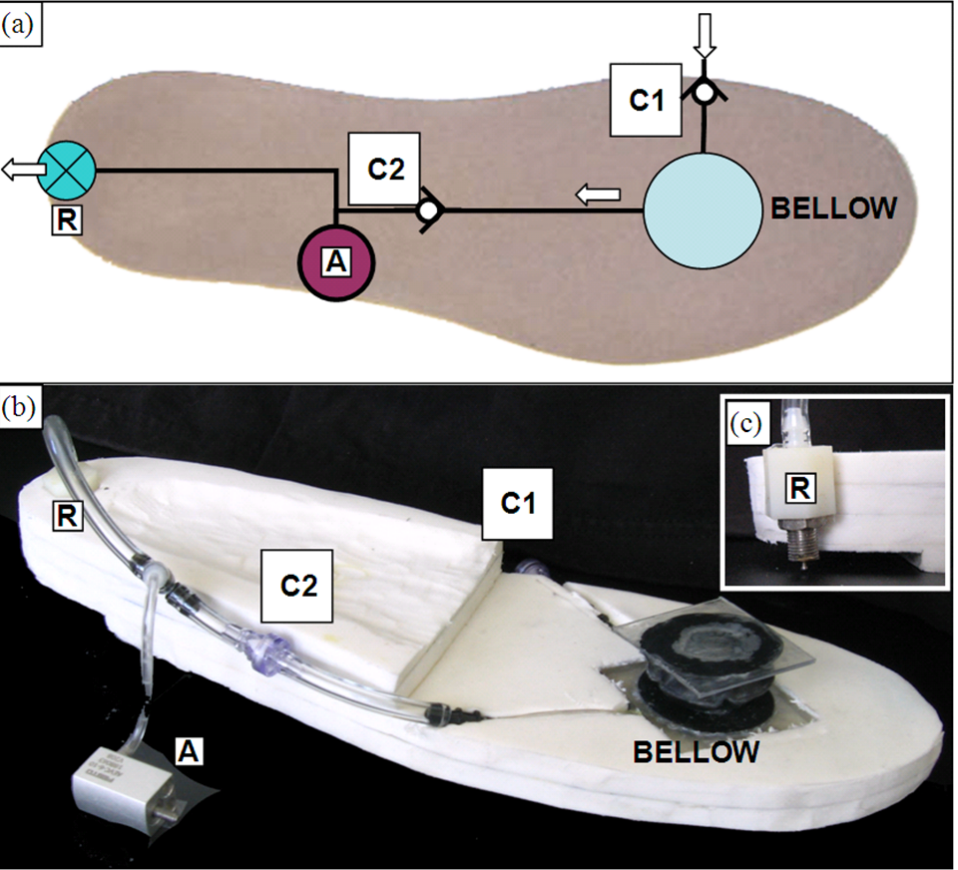
**PhAFO pneumatic circuit diagram and shoe sole**. Pneumatic circuit diagram (a) and actual foam sole (b) with bellow pump mounted at the metatarsal. (C1) check valve is open to atmosphere for intake, (C2) check valve ensures one-way flow direction from bellow to actuator, (A) actuator for locking mechanism, and (R) pressure release valve for exhaust. Valve (R) opens to atmosphere upon contact with the floor (c).

Fluid power was harvested through a pneumatic circuit located between the foot shell and the foam sole (Fig. 2). The sole was comprised of four layers of 7 mm thick sheets of polyethylene foam (Pelite™ Bakelite Xylonite Ltd, Croydon UK). The sole was modified to incorporate the pneumatic circuit for the power harvesting system (which consisted of a bellow pump, valves, and tubing). The pneumatic bellow pump was constructed from a single convolution of a hypalon molded accordion bellow (McMaster-Carr Supply Company, Aurora, Ohio, USA; Model 9421K62) and conical compression spring (McMaster-Carr 1692K31) that were epoxied to two polycarbonate plates. A 3 mm sheet of blue polyethylene foam (MicroPuff Alimed Inc, Dedham, Massachusetts, USA) was used to cover and permit compression and expansion of the bellow (Fig. 1). The design, size and shape of the bellow was based on a recent study [15], which determined the maximum achievable pressure (above 150 kPa) and maximum power generated per gait cycle (peak levels of nearly 10 Watts). A bellow with outside diameter of 4.5 cm was chosen for the present PhAFO since it matched the compressed air volume required by the cylinder and provided the required pressure to activate the actuator cylinder. Details of the available power and the relationship between pressure and cylinder displacement are discussed in [15], where it was noted that Boyle's Law qualitatively described the pressure-time history based on separate tests.

**Figure 3:**
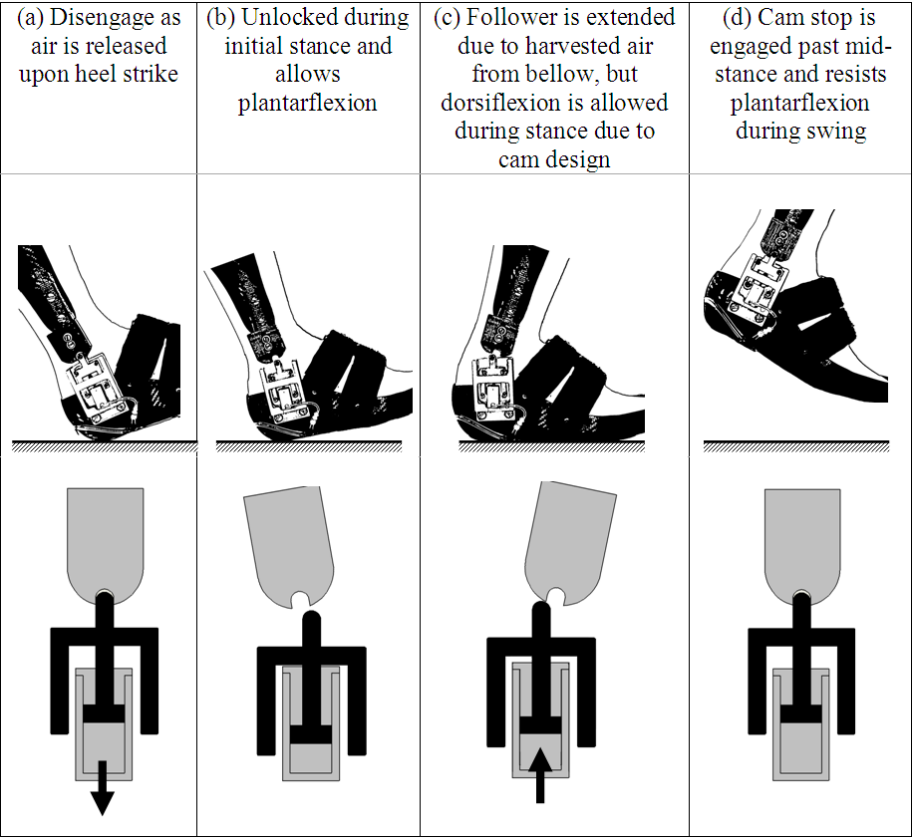
**Ankle joint actuator concept and function during a gait cycle**.

During the stance phase of walking, the weight of the body compressed the bellow and served as the power source for the self-contained pneumatic circuit. This action generated work on the actuated cam-lock mechanism to control plantarflexion during the swing phase. The actuator mechanism consisted of a small linear cylinder with spring return (Festo Corporation, Model AEVC-6-10-A-P), a follower with small rollers, and guide rail housing for the follower (Fig. 1). The minimum pressure required to move the actuator rod was empirically measured to be 120 kPa, similar to the minimum operating pressure specified by the manufacturer. The shape of the follower and guide rail housing prevent potentially damaging shear forces from being transmitted to the cylinder rod. Control and timing of the actuator was accomplished through use of a release valve and the specific placement of the bellow. The bellow was placed under the 2^nd ^and 3^rd ^metatarsal heads. Prior plantar pressure measurements during walking [16,17] and empirical testing determined that this placement achieved the best pressure generation upon bellow compression, while also allowing optimal timing between the release valve and actuator activation. The locking mechanism behavior throughout the gait cycle is detailed in Fig. 3. With the release valve located at the posterior-plantar edge of the AFO's sole, heel strike activates the release valve and allows the compressed air to be discharged from the linear cylinder into the atmosphere (Fig. 3a). At this discharge, the spring in the cylinder retracts the actuator rod to disengage the cam lock and allow free ankle movement (Fig. 3b). As the foot rocks forward during stance, the release valve is closed and fluid power is harvested by compression of the bellow, allowing the pneumatic circuit to be recharged. With compression of the bellow, the cylinder rod extends, simultaneously pushing the follower into extension. Due to the cam design, dorsiflexion during mid-stance is allowed as the follower rolls over the cam surface (Fig. 3c). As the ankle progresses into plantarflexion during late stance, the follower rolls into the cam locking position and secures the foot from plantarflexing beyond the neutral position during swing (Fig. 3d). Note that due to the design of the locking mechanism, when it is engaged, the ankle joint should ideally not be able to plantarflex or dorsiflex away from the neutral position. In essence, both plantarflexion and dorsiflexion are "blocked" once the locking mechanism is engaged, thus holding the ankle joint in the neutral position.

### Biomechanical assessment of PhAFO

The design of the PhAFO was assessed using a pilot study involving an able-bodied control subject (male, 22 years, weight 85 kg, height 174 cm). The subject signed an informed consent form approved by the university institutional review board. Throughout all trials, the subject walked at a self-selected pace on a split-belt instrumented treadmill that has two independent force plates capable of collecting ground reaction force data under each foot (Bertec Corporation, Columbus, Ohio, USA). Average self-selected comfortable walking speed with running shoes was determined after using 0.05 m/s increment and decrements, and identified to be 0.9 m/s.

To assess the influence of the PhAFO on lower limb joint angle behavior, the subject performed two 2-minute walking trials. During the first trial (control), the subject walked in conventional running shoes. During the second trial (PhAFO test condition), the subject wore the PhAFO on the right foot and a conventional running shoe on the left. The heel height of the PhAFO was modified to insure that it was equal to that of the running shoe. Based on our own pilot work, the subject was given five minutes of walking practice to adapt to the PhAFO.

Kinematic data were examined during these trials. For the control trial, reflective markers were attached to the head, torso, arms, and legs. Sagittal plane joint angle data for the ankle, knee and hip were derived using the procedure proposed by Vaughan et al. [18]. For the PhAFO trial, the lateral malleolus, 1^st ^and 5^th ^metatarsal, and heel markers on the right side were removed and markers were placed on the PhAFO to ensure that the motion of the device was tracked correctly. The ankle angle for the PhAFO was computed using the procedure proposed by Winter [19]: two markers were placed collinearly along the lateral aspect of the shank section, a marker was placed lateral to the 5th metatarsal and a marker was placed on the posterior heel region of the PhAFO. To identify the distal end of the shank segment, necessary to compute knee angle, two additional markers were needed since the lateral malleolus marker was removed due to obstruction by the PhAFO. These skin markers were placed on the anterior and posterior surfaces of the distal part of the shank (superior to the ankle joint), such that the distal landmark for the shank segment was defined as the midpoint of these markers. Kinematic data were collected using a six camera infrared motion analysis system sampled at 100 fps (Vicon, Oxford, UK; Model 460). Ground reaction force (GRF) data from the treadmill were sampled at 1000 Hz. Motion and force data were low-pass filtered at 14 Hz and 39 Hz, respectively, using fourth-order, zero-lag, Butterworth filters, determined from the residual analysis method described by Winter [19].

To better examine stance and swing phase mechanics, joint angle data were separated into these two main subdivisions of the gait cycle. The stance phase begins at heel strike and ends at toe-off when that same foot has left the ground. The swing phase then lasts from toe-off until the subsequent heel strike of the same foot. Heel strike was defined as the instance when the vertical GRF exceeded a threshold of 10 N. Toe-off was defined as the instance when the vertical GRF fell below 10 N. The 10 N threshold value was chosen to exceed the noise level of the GRF data. All joint angle data were normalized using the gait events such that subsequent heel strikes were adjusted to 0 and 100% of the gait cycle. Average (and standard deviation) flexion-extension joint angle data for the ankle, knee, and hip of the right side were computed over the entire trial. Due to missing or obstructed markers, 83 and 95 gait cycles were evaluated for the control and PhAFO test trials, respectively. During the 2-minute PhAFO trial, toe clearance was evaluated. For each gait cycle, the minimum distance between the bottom of the PhAFO sole and the treadmill top surface was determined using the vertical position of the marker aligned medial to the 1^st ^metatarsal adjusted by an offset from the marker location to the sole of the PhAFO (48 mm).

To further evaluate repeatable toe clearance during swing and performance of the pneumatic circuit in the PhAFO, the subject performed one 10-minute walking trial while wearing the PhAFO and running shoe combination. GRF and pressure data were collected and sampled at 1000 Hz. Kinematic data could not be collected simultaneously for the 10-minute trial due to limitations of the motion capture system. Vertical GRF measurements were used to verify that there was no foot-ground contact during the swing phase by the limb with the PhAFO during this longer evaluation trial. Custom-made pressure sensing footswitches (constructed from force sensing resistors by Interlink Electronics Inc., Camarillo, California, USA; Model 402, 0.5" circle) were placed in the PhAFO insole under the heel and metatarsal heads. The footswitches were used to examine timing and contact of the fore and rear foot during the gait cycle (heel contact, forefoot contact, and foot-flat). Ground reaction force data is not able to distinguish these discrete foot contact events. A pressure transducer (Setra Systems Inc., Boxborough, Massachusetts, USA; Model 209) was used to record pressure in the pneumatic circuit. The pressure profiles were captured by the pressure transducer connected via a Y-joint to the linear cylinder. Pressure measurements were used to verify pneumatic power generation by the bellows, and actuation/release of the cylinder. GRF and pressure data were processed through a low-pass, second-order, zero-lag, Butterworth filter with 39 Hz and 150 Hz cut-off frequencies, respectively, determined from the residual analysis method described by Winter [19].

## Results

**Figure 4:**
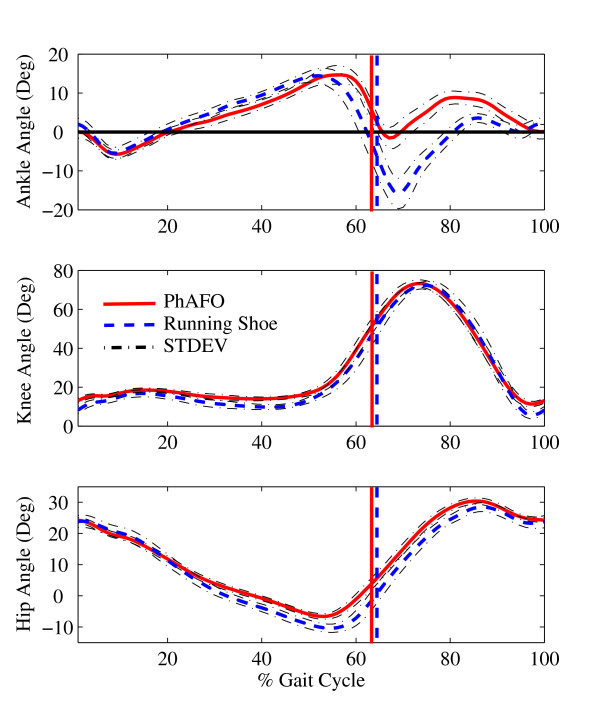
**Ankle, knee, and hip ankle joint range of motion**. Average (and standard deviation) ankle, knee, and hip angular trajectories for the right side of the control subject wearing the PhAFO and normal running shoe over two minute walks. Toe-off for each condition is signified by corresponding vertical line. Positive values = flexion (dorsiflexion).

Kinematic data were used to evaluate the effect of wearing the PhAFO on joint behavior (Fig. 4). The measurements of the ankle angle during the gait cycle were used to quantify the allowance of plantarflexion during initial contact, free dorsiflexion during stance, and the reduction of plantarflexion during swing. As desired, plantarflexion was allowed after initial contact and dorsiflexion was observed through early to mid-stance. As the gait cycle progressed for the control trial, ankle plantarflexion increased with the metatarsal on the ground during the push-off and propulsion phase. However, for the PhAFO gait trajectory, continued plantarflexion during early swing was blocked at the neutral position (0°) as per to the intended design. Unexpected excessive dorsiflexion was observed during mid-swing for the PhAFO trial. The hip and knee trajectories are important measures to determine if excess movements at these joints preempted toe clearance rather than control from the AFO. In general the magnitude and timing of knee and hip joint angles were similar. The timing of the ankle motion was slightly modified by the addition of the PhAFO. A timing difference in reaching peak dorsiflexion was noted between the control and PhAFO conditions. Average timing for the end of the stance phase (or toe-off) was also found to be (a) for the control condition, 64 ± 1% for the right limb and 65 ± 2% for the left; and (b) for the AFO test condition, 63 ± 1% gait cycle for the right limb wearing the PhAFO, and 67 ± 1% for the left.

**Figure 5:**
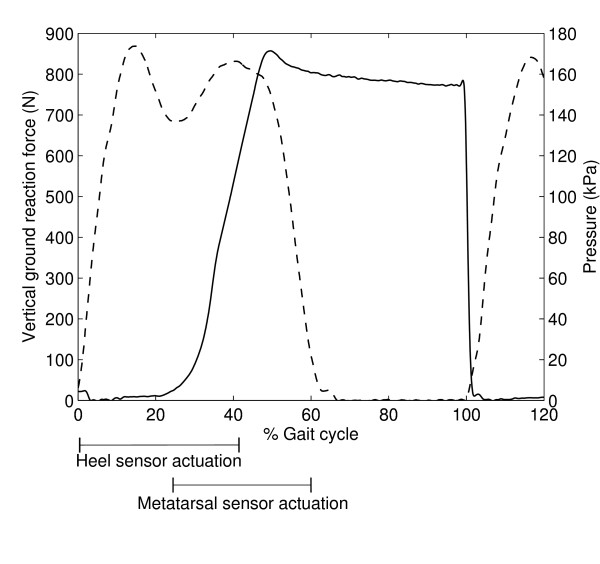
**Ground reaction force and pneumatic pressure**. Sample vertical ground reaction force (dashed line) and pressure measured inline prior to the actuator cylinder (solid line) as a function of percent gait cycle. Heel and metatarsal pressure sensors indicate points of initial contact (heel sensor start), foot flat (overlap of both sensor signals) and toe off (metatarsal sensor end).

Kinematic, foot switch and vertical GRF data were used to assess timing and foot position during stance and to evaluate foot clearance during swing while wearing the PhAFO during level walking. During the initial contact phase (0–2% gait cycle), the foot touches the floor, as shown by the ground reaction force plot and activation of the heel sensor (Fig. 5). With the heel on the ground and acting as a rocker, the ankle plantarflexes into foot flat as noted by the simultaneous activation of both the heel and metatarsal sensors. Stance ends when the foot is completely lifted from the floor. At this point, ground reaction force goes to zero and the metatarsal sensor is deactivated. Ground reaction force remains at zero throughout the remainder of the gait cycle suggesting toe clearance during the swing phase. Kinematic data from the 2-minute PhAFO trial verifies toe clearance between the PhAFO and treadmill surface, which was found to average 33 ± 4 (SD) mm with a minimum observed clearance of 25 mm.

**Figure 6:**
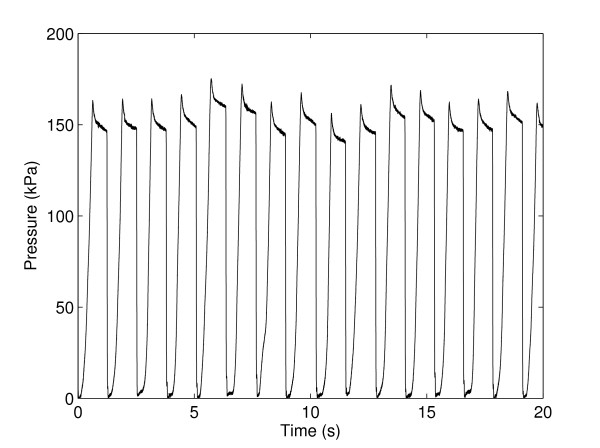
**Multiple step pressure profile**. Pressure profile over multiple gait cycles demonstrating repeated pressure generation from the bellow pump and release at heel strike.

The performance and repeatability of the cyclical locking and unlocking of the cam-lock mechanism during walking was verified. The results showed that the PhAFO was able to generate and discharge air pressure for a 10-minute period without a failure in its functional performance (Fig. 6). The mean maximum pressure was 169 ± 14 kPa. Charge rate of the bellows was determined to be 505 ± 103 kPa/s. The time for building up to maximum pressure was 0.34 ± 0.06 s. After reaching a peak value, pressure decreased gradually prior to release and followed an exponential decay with decay constant 0.10 ± 0.08 s^-1 ^(Fig. 5).

## Discussion

The design, development and testing of a portable power harvesting ankle-foot orthosis that successfully prevents foot-drop was presented (Fig. 1). In particular, the PhAFO has successfully demonstrated effective control of plantarflexion during swing in a normal healthy subject while also permitting free ankle movement during the stance phase of walking (Fig. 4). This view is supported by both kinetic and kinematic measures. Force plate data collected during the 10-minute trial did not record any ground reaction forces during swing, and kinematic data collected during the 2-minute trial found that toe-clearance averaged 33 mm with a minimum clearance of 25 mm. These data demonstrate that no foot-ground contact occurred and adequate toe clearance was achieved (Fig. 5). (Toe clearance during level walking by healthy young and older adults in normal shoes has been found to average 21 ± 5 mm, as measured by a marker over the 1^st ^metatarsal head [20].) Following initial contact, the unlocked ankle joint allowed for full sagittal plane range of motion through stance (plantarflexion – dorsiflexion – plantarflexion) as noted by average ankle angle behavior (Fig. 4). Continued plantarflexion is limited at the neutral position during early swing due to the locking mechanism. Excessive dorsiflexion was observed during mid-swing while wearing the PhAFO. This dorsiflexion may have occurred as a consequence of the able-bodied test subject's desire to compensate for the limited plantarflexion during push-off and unconscious concern to prevent foot-floor contact during swing. This excessive dorsiflexion during swing should not occur with foot-drop patients by virtue of their dorsiflexor weakness. This excessive fluctuation in dorsiflexion during swing, which ideally should not have occurred due to the locking mechanism design, illustrates a need for additional design refinements to limit excess motion in the dorsiflexion direction. Free dorsiflexion during stance was also confirmed using the footswitches placed under the heel and metatarsals (Fig. 5). These switches confirm that both the heel and forefoot were in contact with the ground throughout stance demonstrating that the PhAFO did not force the subject to begin heel rise prematurely.

The capacity of the PhAFO pneumatic circuit to repeatedly harvest fluid power during gait for the operation of the actuated cam-lock mechanism was also validated by the pilot study. During ten minutes of walking, the PhAFO repeatedly pressurized and discharged the pneumatic air circuit, cyclically locking and unlocking the mechanical ankle joint on the orthosis at the desired periods of the gait cycle. The bellow pump consistently generated peak pressures over 150 kPa (Fig. 6), and always exceeded the minimum pressure necessary to activate the linear cylinder, thus providing consistent actuation performance. A small decay in pressure after the maximum pressure was observed and attributed to the volumetric expansion of the bellow as the foot is unloaded from the floor (Fig. 5). The heel-mounted pressure release-valve was found to have a near instantaneous release of pressure upon heel-strike (Fig. 5). In general, PhAFO gait demonstrates motion patterns and ranges of motion for each joint comparable to the non-AFO gait, except for the desired constraint in ankle motion during swing (Fig. 4). Use of the PhAFO resulted in modest timing differences from the control conditions. Peak dorsiflexion was reached later in the gait cycle for the PhAFO trials; however, the general plantar/dorsiflexion behaviors during stance were similar. Stance-swing phase timing was also slightly different. Stance phase tended to be shorter for the side with the PhAFO (63% gait cycle) than the contralateral limb (67% gait cycle) or control gait (64%). Timing was modified such that toe-off occurred as the ankle joint approached the neutral position (0°) during the AFO trial; whereas during the control trial, toe-off occurs after full plantarflexion occurs (approximately -10°). This timing difference is due to the locking of the ankle joint in the neutral position. Further, use of the PhAFO did not substantially modify knee and hip movement and timing behavior. Knee and hip flexion was comparable between conditions; therefore, toe clearance appears to be related to the adequate ankle foot position that the orthosis provided.

A limitation of conventional mechanical ankle joints is that their control mechanisms cannot be timed to the gait cycle to switch function. Thus, a control mechanism that blocks motion often is sustained throughout the gait cycle, thereby sacrificing normal functional movement elsewhere. Such limitation can cause deviations from normal ankle-foot dynamics and could lead to reduced gait stability and efficiency. The proposed PhAFO overcomes this limitation. The design advances demonstrated with the PhAFO are the ability to restrict plantarflexion during swing while permitting free ankle motion during stance, and the capacity to harvest pneumatic power to control these functions. Through use of a mini-actuator to drive a novel cam-lock mechanism, motion control at different times in the gait cycle is possible. The most notable of these advances is the capability for self-power using a pneumatic bellow since this feature allows untethered operation and control of the orthosis. The high forces generated through walking are ideally suited for harvesting fluid power. Multiple channel pneumatic circuits and additional actuators could provide further orthotic control options and features.

Recently, Takaiwa and Noritsugu [21] reported the development of a prototype for another portable pneumatic power-harvesting AFO that supports the foot during swing. The purpose of their design was to reduce falls in older adults by mitigating stumbling associated with shuffling gait. They prevent foot-drop by producing a dorsiflexion-assist moment at the ankle. To do this, their orthotic system used a much larger pneumatic bellow pump located under the heel of a shoe to pressurize a long linear cylinder that powered an actuator. For our study, we elected to use the passive ankle motions during stance phase to engage a compact cam-lock that blocked plantarflexion during swing. Hence, power-assisted dorsiflexion was not necessary. Since this work is a pilot study, this research has several limitations that should be considered when interpreting these findings. Only one able-bodied control subject was used for this initial evaluation. To determine the efficacy of the design, a more thorough clinical investigation using patients diagnosed with foot-drop would be required. As noted, the locking mechanism needs refinement to limit excess motion in the dorsiflexion direction when engaged. Future work is also necessary to examine possible effects of the PhAFO on gait symmetry by examining the contralateral leg behavior when unilateral AFO use is studied. Several design issues that were not undertaken in this project are also limitations. The additional biomechanical control feature to 'dampen' plantarflexion just after initial heel contact was not addressed in this prototype. Another design choice was whether an in-shoe or external system would serve as the platform for the prototype. Since a major objective was to self-harvest power, a prototype design with an outsole was chosen because of its capacity to house the bellow, valve and tubing components compared to the space constraints of an in-shoe type device. Future work is necessary to reduce this design to an in-shoe device.

## Conclusion

The findings from this study demonstrate that fluid power can be harvested with a bellow pump and pneumatic circuit in an AFO to operate a cam-lock mechanism that controls ankle-foot motions at specific periods of the gait cycle. Conceptually, fluid power appears to have potential as a control and power source for use in lower extremity orthotic applications. Considerable research and development is still needed to determine if fluid power can augment and enhance contemporary orthotic systems, and how the technological advantages can be maximized to advance current clinical treatments. The miniaturization of components is an important aspect of future study in this area and is being addressed by our research team.

## Competing interests

An invention disclosure, with intent to obtain a patent in the United States, has been submitted to the University of Illinois at Urbana-Champaign.

## Authors' contributions

All authors contributed to the conception and design of the device. RC developed the bellows concept, constructed the tested device, conducted and analyzed the biomechanical testing data, and drafted the manuscript. ETHW co-supervised the project, assisted with the development and implementation of the biomechanical testing procedure, and helped to draft the manuscript. EL co-supervised the project and helped to draft the manuscript. GK oversaw design of the structural shell and sole, assisted with the development of the biomechanical testing data, and helped to draft the manuscript. SDM developed the cam-locking mechanism. SNT developed the pneumatic circuit and identified valves. KAS assisted with the structural shell, analysis and interpretation of the biomechanical testing data, and editing of the manuscript. JNG modified the sole and cam design, and assisted with device construction and collection and analysis of the biomechanical testing data. All authors read and approved the final manuscript.
